# CABS-dock standalone: a toolbox for flexible protein–peptide docking

**DOI:** 10.1093/bioinformatics/btz185

**Published:** 2019-03-13

**Authors:** Mateusz Kurcinski, Maciej Pawel Ciemny, Tymoteusz Oleniecki, Aleksander Kuriata, Aleksandra E Badaczewska-Dawid, Andrzej Kolinski, Sebastian Kmiecik

**Affiliations:** 1 Biological and Chemical Research Centre, Faculty of Chemistry, University of Warsaw, Warsaw, Poland; 2 Faculty of Physics, University of Warsaw, Warsaw, Poland; 3 College of Inter-Faculty Individual Studies in Mathematics and Natural Sciences, University of Warsaw, Warsaw, Poland

## Abstract

**Summary:**

CABS-dock standalone is a multiplatform Python package for protein–peptide docking with backbone flexibility. The main feature of the CABS-dock method is its ability to simulate significant backbone flexibility of the entire protein–peptide system in a reasonable computational time. In the default mode, the package runs a simulation of fully flexible peptide searching for a binding site on the surface of a flexible protein receptor. The flexibility level of the molecules may be defined by the user. Furthermore, the CABS-dock standalone application provides users with full control over the docking simulation from the initial setup to the analysis of results. The standalone version is an upgrade of the original web server implementation—it introduces a number of customizable options, provides support for large-sized systems and offers a framework for deeper analysis of docking results.

**Availability and implementation:**

CABS-dock standalone is distributed under the MIT licence, which is free for academic and non-profit users. It is implemented in Python and Fortran. The CABS-dock standalone source code, wiki with documentation and examples of use and installation instructions for Linux, macOS and Windows are available in the CABS-dock standalone repository at https://bitbucket.org/lcbio/cabsdock.

## 1 Introduction

Molecular docking of peptides to proteins is often a difficult modelling task. One of the main challenges is modelling conformational flexibility of a protein–peptide system (Ciemny *et al.*, 2018; Geng *et al.*, 2017; Schueler-Furman and London, 2017).

In 2015, we released a web server implementation of the CABS-dock method for flexible protein–peptide docking (Blaszczyk *et al.*, 2016; Kurcinski *et al.*, 2015). Since then, CABS-dock has been used for numerous docking studies, including docking with large-scale conformational transitions of the protein receptor (Blaszczyk *et al.*, 2016; Ciemny *et al.*, 2016). Its range of applicability has been also extended to several advanced modelling protocols allowing residue–residue contact map analysis of the binding dynamics (Ciemny *et al.*, 2017), prediction of protein–protein interaction interface (Ciemny *et al.*, 2017) and docking using information about protein–peptide residue–residue contacts (Blaszczyk *et al.*, 2018).

The main component of CABS-dock is a highly efficient simulation tool: the CABS coarse-grained protein model (Kmiecik *et al.*, 2016; Kolinski, 2004). The CABS model is used for the explicit simulation of protein and peptide flexibility during the search for the binding site (Kurcinski *et al.*, 2015). The simulation tool is combined with modules for models scoring, structural clustering, reconstruction to all-atom representation and analysis of the results. The method offers relatively inexpensive simulations of the backbone flexibility during docking search—docking with default settings (a simulation of fully flexible peptide searching for a binding site on the surface of a flexible protein receptor) usually takes around 24 h for the protein–peptide system with around 1000 residues, using 1 standard CPU.

In this work, we present CABS-dock standalone, which is an upgrade of the web server implementation. It introduces a number of customizable options, provides support for modelling significantly larger protein–peptide systems, and offers a framework for custom result analysis. All options are available from the command line.

## 2 Features

The CABS-dock standalone is distributed as a multiplatform object-oriented Python 2.7 package. The simulation module is implemented in Fortran, while other modules in Python. The package may be installed on Linux, Windows and macOS machines. Additionally, a simple command-line interface is automatically installed together with the package to provide users without any programming background with full control over the procedure.

**Fig. 1.**
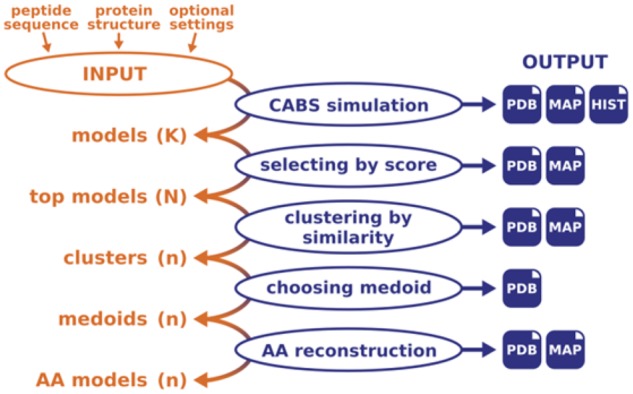
CABS-dock pipeline. The CABS-dock modelling procedure consists of the following steps: CABS simulation (providing K models, default: 10 000); energy scoring (providing N models, default: 1000); structural clustering (providing n clusters, default: 10, default method: *k*-medoids); selecting cluster representatives (providing n models, default selection method: cluster medoids); reconstruction to all-atom (AA) representation [providing n models, default reconstruction method: MODELLER (Webb and Sali, 2014)]. At each modelling step, the number of models may be modified by the user. The default output contains sets of models (generated after each modelling step) in PDB format (in C-alpha representation, or all-atom after the reconstruction procedure), PNG and CSV files with residue–residue contact maps (MAP, for all models, sets of similar models found in clusters and top-scored models) and contact frequency histograms (HIST) for all models generated in CABS simulation

The package is composed of three major modules (see the pipeline in Fig. 1): (i) the CABS simulation module, which combines protein flexibility simulation (using the CABS-flex standalone package (Kurcinski *et al.*, 2018)) and peptide folding and binding into one simulation run; (ii) the scoring module, which selects representative models using energy scoring and structural clustering and (iii) reconstruction of selected models to all-atom representation using the MODELLER package (Webb and Sali, 2014).

As compared with the original web server implementation, the standalone application resolves its most important drawbacks: the necessity of disclosing the modelling data on a public server, limitations regarding the size of the protein–peptide system and limited number of customizable options. In the standalone version, the maximum length of peptide molecules is 50 amino acids (due to the peptide library used for the initial poses generation, this limitation may be overridden by advanced users), while the protein size is primarily limited by the computer memory size (accessible sizes are in the range of thousands of residues). CABS-dock standalone allows customization of every modelling step and provides a number of newly introduced options and tools. New features include tools for generating initial peptide conformations, optional user-defined distance restraints that drive the conformational search towards preferred conformations, settings of conformational flexibility, tools for docking more than one peptide and modification of simulation parameters (among others: temperature and number of Monte-Carlo procedure steps). The package also serves as a comprehensive framework for analysis of simulation results, including calculation of root-mean squared deviation to the reference complex, custom clustering or residue–residue contact frequency maps using user-defined cut-off. The only required input for CABS-dock is the receptor protein structure and the amino acid sequence of the peptide. The receptor may be provided either as a local PDB file or a PDB code.

All the options, examples of use and installation instructions are described in the CABS-dock standalone repository available at https://bitbucket.org/lcbio/cabsdock.

## 3 Conclusions

The CABS-dock standalone application extends the capabilities of the original web server and introduces new features that provide a framework for customizable protein–peptide docking simulations and their analysis. CABS-dock standalone can be particularly useful for simulating large-scale conformational changes of protein–peptide systems and it is well suited for restraint-driven docking. The customizable distance restraints allow the user to impose weak or strong biases towards the desired type of conformations. Thanks to its customizability, modularity and implementation in Python 2.7, CABS-dock standalone (or its modules) may be readily incorporated into existing information-driven docking pipelines (Ciemny *et al.*, 2018; Geng *et al.*, 2017; London *et al.*, 2011; Schueler-Furman and London, 2017; Yu *et al.*, 2017).

## Funding

This work was supported from the National Science Centre (NCN, Poland) Grant [MAESTRO2014/14/A/ST6/00088].

*Conflict of Interest:* none declared.
